# Supplementary material for: Association between hospital liver transplantation volume and mortality after liver re-transplantation
Source: PLoS One. 2021 Aug 5;16(8):e0255655. doi: 10.1371/journal.pone.0255655 (PMC8341477; doi:10.1371/journal.pone.0255655)
Supplement: Attachment — Submitted filename: Response letter_210513.docx [file pone.0255655.s001.docx]

**Response letter regarding manuscript PONE-D-20-31343 entitled “Association between hospital liver transplantation volume and mortality after liver re-transplantation"**

Dear Editor and Reviewers

First of all, we would like to express our gratitude for the constructive comments by the reviewers that has helped us significantly improve the quality of our manuscript. And we sincerely appreciate the extension of the deadline for submitting revisions even though the waiting time for reanalysis was long due to the priority of COVID-19 related analysis. As we went through the comments and questions, we were grateful for the time and effort the reviewers have obviously made to point out important aspects. The following are the response to the reviewers’ comments/questions, point per point.

***Reviewer #1’s comments***

*This retrospective study analyzed national insurance data regarding liver re-transplantation in the Republic of Korea (a.k.a. South Korea). It is a well written and interesting manuscript, however some points need clarification.*

*1. Was there a significant difference in the incidence of re-transplantation between high-volume and low-volume centers (defined as performing <64 or >= 64 LT annually)? Background: the incidence of re-transplantation may also be seen as a performance indicator of a center especially with regard to incidence of primary non-function (PNF), small-for-size-syndrome (SFSS), or early Hepatic artery thrombosis (HAT).*

- Thank you for your kind comment. Considering that primary non-function, small-for-size syndrome, and early hepatic artery thrombosis are all indications for early re-transplantation, the incidence of early re-transplantation would be more appropriate as a performance indicator than the incidence of all re-transplantation. As you commented, we calculated the incidence of liver re-transplantation in the high-volume and low-volume centers.
- During the study period, the number of primary LTs was 6027 cases in high volume centers and 4684 cases in low volume centers. The incidence of liver re-transplantation was higher in high-volume centers (2.9% [175/6027] vs. 1.8% [83/4684], P < 0.001) than in low-volume centers. However, the incidence of early re-transplantation was lower in the high-volume than in low-volume centers (0.8% [51/6027] vs. 1.0% [45/4684]).
- High volume centers showed a higher proportion of living donor LT (78.7% [4174/6027] vs. 69.0% [3232/4684]), which is considered to be more challenging due to insufficient graft volume and the smaller diameter of the blood vessels requiring anastomosis than deceased donor LT compared to low-volume centers.
- Lower incidence of early re-transplantation despite the higher proportion of living donor LT in high-volume centers compared to low-volume centers can be interpreted as a better performance level of high-volume centers, and these findings are consistent with the results of this study supporting the centralization of liver re-transplantation. However, due to the lack of information on the specific cause of early re-transplantation, it cannot be generalized that these values reflect the surgical quality of the centers. We added several sentences in the 5^th^ paragraph of the ‘Discussion’ section and several variables in the table 1 as follows.”

**“In addition, despite the higher proportion of living donor LT, which is more challenging than deceased donor LT, the high-volume centers showed lower incidence of early re-transplantation compared to low-volume centers. Since the incidence of early re-transplantation due to primary non-function, small-for-size syndrome, or early hepatic artery thrombosis after LT can be considered as a performance indicator, this result may be another piece of evidence supporting centralization of liver transplantation.”**

**Table 1. Patient characteristics**

|  | Low-volume center  (< 64 LTs/year) | High-volume center  (≥ 64 LTs/year) | *P*-value |
| --- | --- | --- | --- |
| Donor type of re-transplantation |  |  | 0.549 |
| Living donor | 14 (16.9) | 35 (20.0) |  |
| Deceased donor | 69 (83.1) | 140 (80.0) |  |
| Total number of primary LTs | 4684 | 6027 |  |
| Proportion of re-transplantation |  |  | < 0.001 |
| Early re-transplantation | 1.0 | 0.8 |  |
| Late re-transplantation | 0.8 | 2.1 |  |

*2. Was there any difference between high-volume and low-volume centers regarding donor source (i.e. DBD vs. DCD vs. LDLT?)*

- Thank you for your comment. Since donation after circulatory death (DCD) is currently not a viable option in Korea, all grafts are either donation after brain death (DBD) or living donor.
- We added the results regarding the difference between the high-volume and low-volume centers in the donor source of re-transplantation in table 1 as follows. There was no statistically significant difference between the two groups.

**Table 1. Patient characteristics**

|  | Low-volume center  (< 64 LTs/year) | High-volume center  (≥ 64 LTs/year) | *P*-value |
| --- | --- | --- | --- |
| Donor type of re-transplantation |  |  | 0.549 |
| Living donor | 14 (16.9) | 35 (20.0) |  |
| Deceased donor | 69 (83.1) | 140 (80.0) |  |

- As new variables were added, the results of the multivariate analysis conducted including this variable were also changed. The third, fourth, and fifth paragraph of the “Results” section and table 2, 3, and 4 were revised as follows.

**“After adjustment, 60 years or older (OR 2.39, 95% CI [1.02, 5.60], *P* = 0.044), female (OR 2.05, 95% CI [1.14, 4.10], *P* = 0.032), liver re-transplantation in low-volume centers (OR 1.93, 95% CI [1.00, 3.73], *P* = 0.050), hepatitis A infection (OR 4.15, 95% CI [1.09, 15.84], *P* = 0.037), graft from deceased donor (OR 7.75, 95% CI [2.13, 28.23], P = 0.002), and liver re-transplantation between 2011 and 2013 (OR 2.11, 95% CI [1.03, 4.34], *P* = 0.042) were identified as risk factors of in-hospital mortality after liver re-transplantation (Table 2).”**

**“The overall 1-year mortality rate after liver re-transplantation was 36.8% (81/220); 32.2% (48/149) in high-volume centers and 46.5% (33/71) in low-volume centers (*P* = 0.041). Low-volume centers showed a significantly higher 1-year mortality compared to high-volume centers (OR 2.54, 95% CI [1.24, 5.21], *P* = 0.011) after adjusting for relevant factors. In addition to case volume, older age (≥ 60 years), presence of hepatitis C virus, graft from deceased donor, and liver re-transplantation before 2014 were identified as significant risk factors of 1-year mortality after liver re-transplantation (Table 3).”**

**“Evaluation of long-term survival for up to 9 years after liver re-transplantation showed lower survival in patients who received liver re-transplantation in low-volume centers compared to high-volume centers (*P =* 0.002) (Figure 3). Multivariable Cox regression analysis also showed a higher mortality rate in low-volume centers (adjusted HR 2.12, 95% CI [1.37, 3.27], *P* < 0.001) compared to high-volume centers (Table 4). The discrepancy in survival rates between high and low-volume centers gradually increased to about 10% at 1 year after liver re-transplantation and was maintained thereafter.”**

*3. The age grouping is confusing, it would be better to replace it with median recipient age and range.*

- Thank you for your thoughtful comment. Median age of recipients in low-volume and high-volume centers were 54 years and 52 years, respectively (*P* = 0.893). As recommended, the age in Table 1 was revised with median recipient age and range as follows.

**Table 1. Patient characteristics**

|  | Low-volume center  (< 64 LTs/year) | High-volume center  (≥ 64 LTs/year) | *P*-value |
| --- | --- | --- | --- |
| Age | 54 (23, 68) | 52 (19,74) | 0.893 |

*4. The relationship between timing of re-transplantation and outcome needs a discussion that is even more nuanced and differentiating. The AU themselves point out that early re-transplantation is often technically easier, however graft quality may be compromised (since it is mostly an emergency procedure that has to be carried out a.s.a.p - whereas late re-LT is often an elective procedure), furthermore due to the emergency character patients are often in critical care with multi-organ failure. Now add to this that the need of early re-transplantation may a consequence of errors in judgement and/ or performance (PNF, SFSS, HAT see above) it comes as no surprise that early re-transplants occur more often at low-volume centers and have a worse outcome. Referral of a patient in this scenario from a low-volume to a high-volume center does not seem realistic and probably would not change the outcome. However what may follow from these data would be a recommendation of referral of late re-transplants to high-volume centers.*

- Thank you for your constructive comment. We are of the same opinion and several sentences were added to the 5^th^ paragraph of the ‘Discussion’ section as follows.

**“However, it cannot be generalized that all re-transplantations should be performed in high-volume centers. The cause of early re-transplantation may be technical issues, but it may also be misjudgment. Therefore, early re-transplantation may occur more frequently in inexperienced low-volume centers compared to high-volume centers. Since most early re-transplantations are performed in critically ill patients with rapidly progressing hepatic failure, transplantations must be performed as quickly as possible, and transferring patients to high-volume centers may be risky. Therefore, centralization may be more suitable for late re-transplantations in which the procedure may be performed in an elective basis.”**

***Reviewer #2’s comments***

*I think this is an interesting and original retrospective study evaluating the impact of centre volume on the outcome of liver re-LT. The main limit of this is well underlined by the authors in the discussion and it is the absence of some relevant variables in the analysis (i.e. MELD score, HCC characteristics). I have only some minor comments.*

*1. The authors found 64 as cut off to define high and low volume centers using ROC curve. However, it is not clear to me what was the endpoint of the ROC curve? It was in hospital mortality, or 1-year mortality, or other?*

- Thank you for your comment. The endpoint of the ROC curve was in-hospital mortality. To clarify, the second sentence of the paragraph that has the subheading titled ‘Definition of case volume’ in the ‘Materia and Methods’ section was revised as follows.

**“To determine a cut-off for dividing low and high-volume centers, receiver operating characteristic (ROC) curve analysis between institutional case volume and in-hospital mortality was performed.”**

*2. Reading the Methods section it seems that surgical technique of first LT and re-LT (LDLT vs. DDLT) probably is available for the authors. Why did not include this variable in logistic and Cox regressions?*

- Thank you for your comment. As suggested, the results of the additional analysis regarding the difference between high-volume and low-volume centers in the donor source of re-transplantation was added to table 1 as follows. There was no statistically significant difference between the two groups.

**Table 1. Patient characteristics**

|  | Low-volume center  (< 64 LTs/year) | High-volume center  (≥ 64 LTs/year) | *P*-value |
| --- | --- | --- | --- |
| Donor type of re-transplantation |  |  | 0.549 |
| Living donor | 14 (16.9) | 35 (20.0) |  |
| Deceased donor | 69 (83.1) | 140 (80.0) |  |

- As a new variable was added, the results of the multivariate analysis conducted sere revised. The third, fourth, and fifth paragraph of the “Results” section and table 2, 3, and 4 were changed as follows.

**“After adjustment, 60 years or older (OR 2.39, 95% CI [1.02, 5.60], P = 0.044), female (OR 2.05, 95% CI [1.14, 4.10], P = 0.032), liver re-transplantation in low-volume centers (OR 1.93, 95% CI [1.00, 3.73], P = 0.049), hepatitis A infection (OR 4.15, 95% CI [1.09, 15.84], P = 0.037), early re-transplantation (OR 1.26, 95% CI [0.65, 2.45], P = 0.049), graft from deceased donor (OR 7.75, 95% CI [2.13, 28.23], P = 0.002), and liver re-transplantation between 2011 and 2013 (OR 2.11, 95% CI [1.03, 4.34], P = 0.042) were identified as risk factors of in-hospital mortality after liver re-transplantation (Table 2).”**

**“The overall 1-year mortality rate after liver re-transplantation was 36.8% (81/220); 32.2% (48/149) in high-volume centers and 46.5% (33/71) in low-volume centers (P = 0.041). Low-volume centers showed a significantly higher 1-year mortality compared to high-volume centers (OR 2.54, 95% CI [1.24, 5.21], P = 0.011) after adjusting for relevant factors. In addition to case volume, older age (≥ 60 years), presence of hepatitis C virus, graft from deceased donor, and liver re-transplantation before 2014 were identified as significant risk factors of 1-year mortality after liver re-transplantation (Table 3).”**

**“Evaluation of long-term survival for up to 9 years after liver re-transplantation showed lower survival in patients who received liver re-transplantation in low-volume centers compared to high-volume centers (P = 0.002) (Figure 3). Multivariable Cox regression analysis also showed a higher mortality rate in low-volume centers (adjusted HR 2.12, 95% CI [1.37, 3.27], P < 0.001) compared to high-volume centers (Table 4). The discrepancy in survival rates between high and low-volume centers gradually increased to about 10% by 1 year after liver re-transplantation and was maintained thereafter.”**

- We also conducted an analysis of the donor source of primary LT. However, data were not available for 27.5% of patients who underwent primary LT before 2007 and therefore could not be included in the multivariate analysis.

|  | Low-volume center  (< 64 LTs/year) | High-volume center  (≥ 64 LTs/year) | *P*-value |
| --- | --- | --- | --- |
| Donor type of primary LT |  |  | 0.062 |
| Living donor | 42 (50.6) | 76 (43.4) |  |
| Deceased donor | 26 (31.3) | 43 (24.6) |  |
| Unknown | 15 (18.1) | 56 (32.0) |  |

I would like to thank the editor and the reviewers once again for their constructive comments and suggestions, which have significantly improved the quality of our paper.

Sincerely,

Ho Geol Ryu M.D. Ph.D.

Department of Anesthesiology and Pain Medicine, Seoul National University Hospital,

Seoul National University College of Medicine,

101 Daehak-ro, Jongno-gu, Seoul, Korea, 03080

Tel: 82-2-2072-2065, Fax: 82-2-747-5639, E-mail: hogeol@gmail.com
